# Dermatitis Caused by Plants

**Published:** 1902-08-16

**Authors:** 


### DERMATITIS CAUSED BY PLANTS.

It is well known that the leaves of certain plants produce upon the skin peculiar and very irritating effects. Perhaps the best known instance is that of the *primula obconica*, contact with which is apt to produce a condition of erythema, which is not only very annoying but has often been the cause of much anxiety when its origin has not been suspected. Dr. Hearnden,[1] of Leatherhead, has related a case in which severe dermatitis was caused by the leaves of *Humea elegans*, a plant which is a native of New South Wales, and is now frequently grown in greenhouses for the sake of its perfume. The leaves are not unlike those of the tobacco plant in shape, but they are much more shiny, and exhale a strong smell like incense. On rubbing a leaf on his arm a gummy secretion, with a powerful smell, was left, and a bright red punctiform rash followed, which lasted all day but caused little irritation. A gardener, however, told him that he had noticed his arm itching after moving the plants, and also that the gardener who had given him the plants had suffered from a severely inflamed face, which was ascribed to an irritating soap, but was no doubt due to the *humea*. The case which attracted attention to the subject was one in which the patient, a female, who had a particularly delicate complexion, had been frequently suffering during the last few months from vesicular eruptions on the nose and cheeks looking like impetigo. They quickly subsided under treatment, but as there was an almost immediate relapse she consulted a specialist who diagnosed eczema and advised arsenic. On seeing her, Dr. Hearnden found a red swollen face, the left eye closed, large vesicles containing clear serum on the cheeks, and a patch of eczema on the left side of the chin. The attack subsided, but on going out at the end of a week it returned again as badly as before. Now for the explanation. She had picked a leaf of *Humea elegans* and had smelt it; moreover, it appeared that during the last few months she had been in the habit of picking a leaf and rubbing it on her veil as she liked the perfume! The position of the rash was just where the veil would touch the face, and the patch on the chin corresponded to the place where the veil was screwed up and tucked in.

[1] Lancet, July 26.

